# The Role of PARP Inhibitors in the Ovarian Cancer Microenvironment: Moving Forward From Synthetic Lethality

**DOI:** 10.3389/fonc.2021.689829

**Published:** 2021-06-14

**Authors:** Margherita Turinetto, Giulia Scotto, Valentina Tuninetti, Gaia Giannone, Giorgio Valabrega

**Affiliations:** ^1^ Department of Oncology, University of Torino, Torino, Italy; ^2^ Candiolo Cancer Institute, FPO-IRCCS, Candiolo, Italy

**Keywords:** PARP inhibitors, ovarian cancer, tumor microenvironment, immune checkpoint inhibitors, immune system response

## Abstract

PARP inhibitors (PARPi) have shown promising clinical results and have revolutionized the landscape of ovarian cancer management in the last few years. While the core mechanism of action of these drugs has been largely analyzed, the interaction between PARP inhibitors and the microenvironment has been scarcely researched so far. Recent data shows a variety of mechanism through which PARPi might influence the tumor microenvironment and especially the immune system response, that might even partly be the reason behind PARPi efficacy. One of many pathways that are affected is the cGAS-cGAMP-STING; the upregulation of STING (stimulator of interferon genes), produces more Interferon ϒ and pro inflammatory cytokines, thus increasing intratumoral CD4+ and CD8+ T cells. Upregulation of immune checkpoints such as PD1-PDL1 has also been observed. Another interesting mechanism of interaction between PARPi and microenvironment is the ability of PARPi to kill hypoxic cells, as these cells show an intrinsic reduction in the expression and function of the proteins involved in HR. This process has been defined “contextual synthetic lethality”. Despite ovarian cancer having always been considered a poor responder to immune therapy, data is now shedding a new light on the matter. First, OC is much more heterogenous than previously thought, therefore it is fundamental to select predictive biomarkers for target therapies. While single agent therapies have not yielded significant results on the long term, influencing the immune system and the tumor microenvironment *via* the concomitant use of PARPi and other target therapies might be a more successful approach.

## Background and State of the Art

Over the past decade, poly(ADP-ribose) polymerase inhibitors (PARPi) have completely revolutionized the landscape of Ovarian Cancer (OC) treatment. The phase III trials [NOVA (1), SOLO2 (2), and ARIEL3 (3)] conducted for PARPi as maintenance treatment after platinum-based chemotherapy have shown remarkable results in terms of progression free survival PFS, and while the SOLO2 long term analysis didn’t show an improvement in OS that was statistically significant, it was interpreted as clinically significant (2). This is incredibly important when considering the lack of alternatives to chemotherapy aforementioned and the tolerable and manageable side effects demonstrated by PARPi.

Currently, PARPi have been implemented in the clinical standards as maintenance therapy in first line BRCA mutated high grade serous ovarian cancer (HGSOC) stages III and IV after partial or complete response to platinum salts, in II line and onward for platinum sensitive relapsed high grade serous or endometrioid OC (4).

While the association between chemotherapy and PARPi has been proven to be burdened by greater toxicities, there are currently a number of trials focusing on PARPi in other scenarios; in the single agent setting [ARIEL4 for Rucaparib (5) and SOLO3 for Olaparib (6)], and in association with other target therapies such as Cediranib, an anti VEGFR [ICON9 (7)] or ICI such as Nivolumab (ATHENA) (8).

Despite the efforts made, immunotherapy is still in the sidelines when it comes to the treatment of OC, at the moment considered a poor responder.

Even if the biology of the tumor suggests that patients might benefit from immunotherapy, the clinical data is not as promising as expected (9).

The use of single agents such as anti PDL1 or anti CTLA4 showed a median response rate (RR) of just 10–15% (10).

In the JAVELIN PARP 200, patients with platinum refractory or resistant OC were treated with PLD or Avelumab single therapy, or Avelumab + PLD, and there was no significant improvement of PFS or OS in the experimental arms compared to PLD alone.

So far combination regimens have not showed any significant benefit; in the phase III IMAGYN050 patients affected by stage III/IV OC were randomized to receive Carboplatin-Taxol-Bevacizumab + Atezolizumab or placebo. The progression free survival (PFS) in the intention to treat (ITT) population did not show any difference in the two cohorts (18.4 months with placebo *vs* 19.5 months with Atezolizumab), overall survival (OS) seems to be similar as well, even though the results are still maturing (11).

The JAVELIN PARP 100 enrolled patients with advanced or metastatic OC, treating the population with Carboplatin-Taxol + Avelumab or placebo, followed by maintenance therapy with Avelumab and Talazoparib. It was discontinued after an interim data analysis indicated the PFS primary endpoint would not be met (12).

There is increasing evidence for their important role in modifying tumor microenvironment (13).

Besides HRD, replication fork instability is another major mechanism through which PARPis are effective, because PARP1 is implicated in fork protection, cooperating with BRCA2 (14). In the absence of restored HR, the replication fork stability has recently been identified as a resistance mechanism (15).

As we will discuss later on, this may be relevant for combining PARPi with immunotherapy (16).

Given the increased T cell inflammation in HRD positive tumors as well as preclinical studies showing the synergistic anti-tumor effects of PARP inhibitors and CTLA or PD(L)1 blockade in EOC carrying BRCA mutations (17) several trials exploring the same combination are ongoing in BRCA mutated and BRCA wild-type patients, respectively (NCT02571725; NCT02485990; NCT02953457).

In this review we will discuss how the effect of PARPi on the tumor environment might boost response to ICI.

## BRCA: Different Immune-Modulatory Effect at Genomic Level of BRCA1 Mutation Compared to BRCA2

Although the majority of OC are sporadic (18), a pathogenic mutation in either BRCA1 or BRCA2 confers respectively 36 to 60% and 16 to 27% (19). Loss of BRCA1 function has also been documented through its promoter methylation in about 10% of high grade serous ovarian cancer (HGSOC) patients. Although to a lower extent, alterations in other genes of the homologous DNA repair pathway like ATM, ATR and Fanconi anemia genes can also cause DNA homologous repair deficiency (HRD) (20). Epigenetic silencing of BRCA1 and RAD51C expression through DNA hypermethylation also results in a DNA homologous repair deficiency (HRD) (21) (22) (23).

BRCA1/2-mutated HGSOCs exhibit a higher mutational load and a unique mutational signature thus harboring more tumor-specific neoantigens that stimulate recruitment of an increased number of tumor-infiltrating lymphocytes (TILs), which is counterbalanced by overexpression of immune checkpoints such as PD-1 or PD-L1.

While both genes have pleiotropic effects on the genesis of the tumor, recent data highlights that they affect in different manners the tumorigenesis, the microenvironment, and the immune response, resulting in contradistinctive mutational landscapes.

Samstein et al. analyzed this in murine models how the two mutations differ in their functional impact on the tumor microenvironment. BRCA2 mutation has a known core function in homologous recombination (HR), one of the most commonly altered DNA damage pathways in cancer, which results in a higher tumor mutational load. Another indicator of genetic instability, mismatch repair deficiency (MRD) has already been approved by FDA as a predictive marker for response to ICI (24); the lack of success of immunotherapy in OC may therefore be a result of our incomplete knowledge of other factors influencing the microenvironment and the immune response rather than a failed therapy approach.

Further analysis of the BRCA2mut murine orthotopic breast cancer cell line compared to a syngeneic BRCA2wt line showed increased CD4+ and CD8+ in tumor infiltrating lymphocytes, NK mediated cytotoxicity, *α*-interferon, and cytokine signaling. Angiogenesis and epithelial to mesenchymal transition (EMT) were also influenced. When exposed to ICI, specifically anti PD1 or anti CTLA4 and anti PD1, the BRCA2mut mammal tumor exhibited a greater response, with significant growth delay, compared to the BRCA2wt cell line. This was not the case with the syngeneic BRCA1 mut murine model; the difference in elicited response to ICI is the result of a dissimilar effect in both adaptive and innate immune activation, further proven by direct examination between BRCA1 and BRCA2 mut yielded tumor programs.

BRCA1 mutation also showed an upregulation of immunosuppressive and regulatory genes, while BRCA2 mutation had an increased expression of immune cell activation genes.

In recent preclinical mouse model studies, it was observed that also PI3K pathway inhibition resulted in genomic instability, and cancer cells with reduced activity in this pathway are more sensitive to PARP inhibition (25, 26). These results have prompted investigators to test the combination of PI3K and PARP inhibitors in OC (27); PI3K, mTORc1/2, and AKT selective inhibitors have been tested with Olaparib (28).

These results, provided the preclinical aspect and the lack of EOC models, may give an insight on a more heterogeneous landscape of responses to both PARPi and ICI in OC, that requires the identification of reliable predictive markers (29).

## PARP Role in the Immune System

In murine models, the T cell development has been shown to require PARP efficiency in all its stages; PARP2 deficiency decreases the number of thymocytes, causing a higher number of apoptosis during the maturation process. PARP1 and 2 deficiency causes a reduction in both CD4+ and CD8+ peripheric population (30).

PARP is also involved in cell activation; PARP1 deficiency seems to direct T cells to the Th1 (31) and Treg phenotype rather than Th2 (32).

B lymphocytes are affected as well, although the reasons are not yet completely clear; dual deficiency affects the peripheric population is the same way it does with T cells, proving to be consistent with the hypothesis that PARP preventing the accumulation of DNA damage is needed during cell proliferation.

PARP plays a role in the activation and recruitment of neutrophils (33), the expression of pro inflammatory cytokines such as IL-6, TNF by macrophages (34) and the recruitment of dendritic cells (in which PARP1 is involved, but PARP2’s role remains unclear) (35).

The effects of PARP inhibition are therefore complex and overlapping.

## PARP Inhibitors Effects: Moving Forward From Synthetic Lethality

### Hypoxia

Solid tumors display different levels of oxygenation, especially when characterized by a high cellular replication. It is possible to identify areas where angiogenesis is not able to provide enough oxygen for the quickly growing mass, which will cause varying degrees of hypoxia, from chronic low levels to severe. It’s widely known how this characteristic has a negative prognostic impact, because it is a sign of fast proliferation of the tumor and because it is linked to resistance to chemotherapy, radiotherapy, decrease of DNA repair, and increase of the risk of metastatic spreading.

Both severe and chronic hypoxia cause a disruption in the HR pathway, vital for DNA double strand breaks repair, by decreasing the translation of proteins like RAD51, BRCA1, and BRCA2 (36); Chan et al. (37) confirmed this theory *via* the observation of RAD51 activity in hypoxic cells in murine models; RAD51 remained suppressed even at chronically moderate levels of hypoxia. Notably, this was the first mechanism observed that was independent from hypoxia inducing factor 1 alfa (HIF1alfa). It was afterwards theorized how HR defective hypoxic cells could be sensitive to PARPi, irrespective of their BRCA status, which was proven true in various cell lines. The most effective result was observed in cells subjected to severe hypoxia, followed by reoxygenation, probably because of the synergistic effect of reactive oxygen species (ROS) toxicity and PARP inhibition.

Thus far PARPi have been considered effective in BRCA mutated and HRD defective cells; the new concept of contextual synthetic lethality, whereby external conditions such as hypoxia can alter the tumor cell mechanisms rendering them susceptible to target therapies, is surely worth exploring further and can offer new scenarios for clinical setting that were previously thought to be immune to PARPi (38).

Aiming at creating contextual synthetic lethality within the tumor microenvironment, several studies are testing the effect of anti-angiogenic therapy in combination with PARP inhibitors [i.e. PAOLA1 (39) and AVANOVA2 (40) with already available results, MITO25 (41) and CONCERTO (42) currently ongoing].

### STING Pathway

There is increasing evidence that the interaction between DNA damage and the immune system plays an important role in the success of cancer treatment. One of many pathways that are affected is the cGAS-cGAMP-STING; the presence of pathogenic DNA elicits the upregulation of STING (stimulator of interferon genes), thus producing more Interferon ϒ and pro inflammatory cytokines.

Ding et al.’s work delves further into this concept, using murine models engrafted with high grade serous ovarian cancer with concurrent loss of p53 and Brca1 and overexpression of c-Myc. Treatment of these models with Olaparib showed significant responses, which were proven to be also driven by the host immune response because they were significantly less noticeable when introducing an anti CD8 antibody or other means of immune suppression.

Specifically, it was observed that Olaparib increased intratumoral CD4+ and CD8+ cells, decreased the production of inhibitory receptors, increased the recruitment and activity of tumor associated dendritic cells, therefore providing a robust immune activation both innate and adaptive.

The importance of STING upregulation for the immune stimulating activity of Olaparib was proven by the significant reduction of response observed in STING knock out mice (43).

The STING pathway was also the focus of Shapiro et al.’s paper. BRCA1 deficient triple negative breast tumor cells were engrafted in mice models; through the activation of the STING pathway, Olaparib significantly increased CD3+ and CD8+ T cells. Granzyme B in CD8+ cells and in NK was also increased, indicative of cytolytic action. While CD4+ T cells were incremented, the Treg phenotype was not found to be more expressed, thus suggesting a higher T helper differentiation. mRNA levels of INFβ, CCL5, and CXCL10, potent proinflammatory signals that correlate to T cell infiltration, were found to be overexpressed. This was not the case in a BRCA1 proficient setting. These findings suggest that PARPi is a potent inductor of both cytotoxic cells recruitment and activation, but the effect is a lot more prominent in a HRD deficient or BRCA mutated models (44, 45).

### Upregulation of PDL-1

Immune checkpoints are now recognized to be of fundamental importance in carcinogenesis; a wide range of tumor cells express both PDL1 and 2, and their overexpression contributes to impairment of multiple signaling pathways of the adaptive immune system (46).

An upregulation of PD-L1 after Olaparib treatment in the murine models was also detected in Dyng et al.’s research; while anti PD-L1 alone provided little to no response, combination of Olaparib and PD-L1 showed significant effect (43).

A similar mechanism was studied by Shen et al., through a model that differed from Dyng’s in regard to HR deficiency; in a HR proficient setting, Talazoparib was used in synergy with anti PDL1, observing once again an increased level of PDL1 and a significant response to the combination (47).

Besides indirect stimulation through immune signaling, PARPi may induce upregulation of PD-L1 levels through inactivation of GSK3β, which was further proven in Jiao’s work by the lack of increase in PD-L1 in GSK3β knock out murine models when treated with Olaparib (48). The hindering of T cell killing caused by higher PD-L1 and PD1 levels seems to reverse when PARPi therapy is combined with ICI.

While expression of PD-L1 on tumor cells is a predictive biomarker of response to ICIs in other cancer types (i.e., NSCLC and urothelial cancers), its expression in EOC is not very frequent (10–33%) suggesting that not all tumors rely on this pathway for immune evasion and its prognostic role in EOC is still controverted. Some data suggest that expression of PD-L1 correlates with worse prognosis (49), while in other series a higher expression correlates with better PFS (50).

In the TOPACIO trial, exploratory analyses of subpopulations did not reveal any difference in clinical activity of the duet Pembrolizumab + Niraparib between PD-1 high and PD-1 low tumor status, thus not indicating PD-1 level of expression as a predictive biomarker (51).

## Discussion

PARPi have undoubtedly revolutionized the landscape of OC cancer, but new treatments need to be explored as up to 70% of advanced OC eventually relapse.

**Figure 1:**
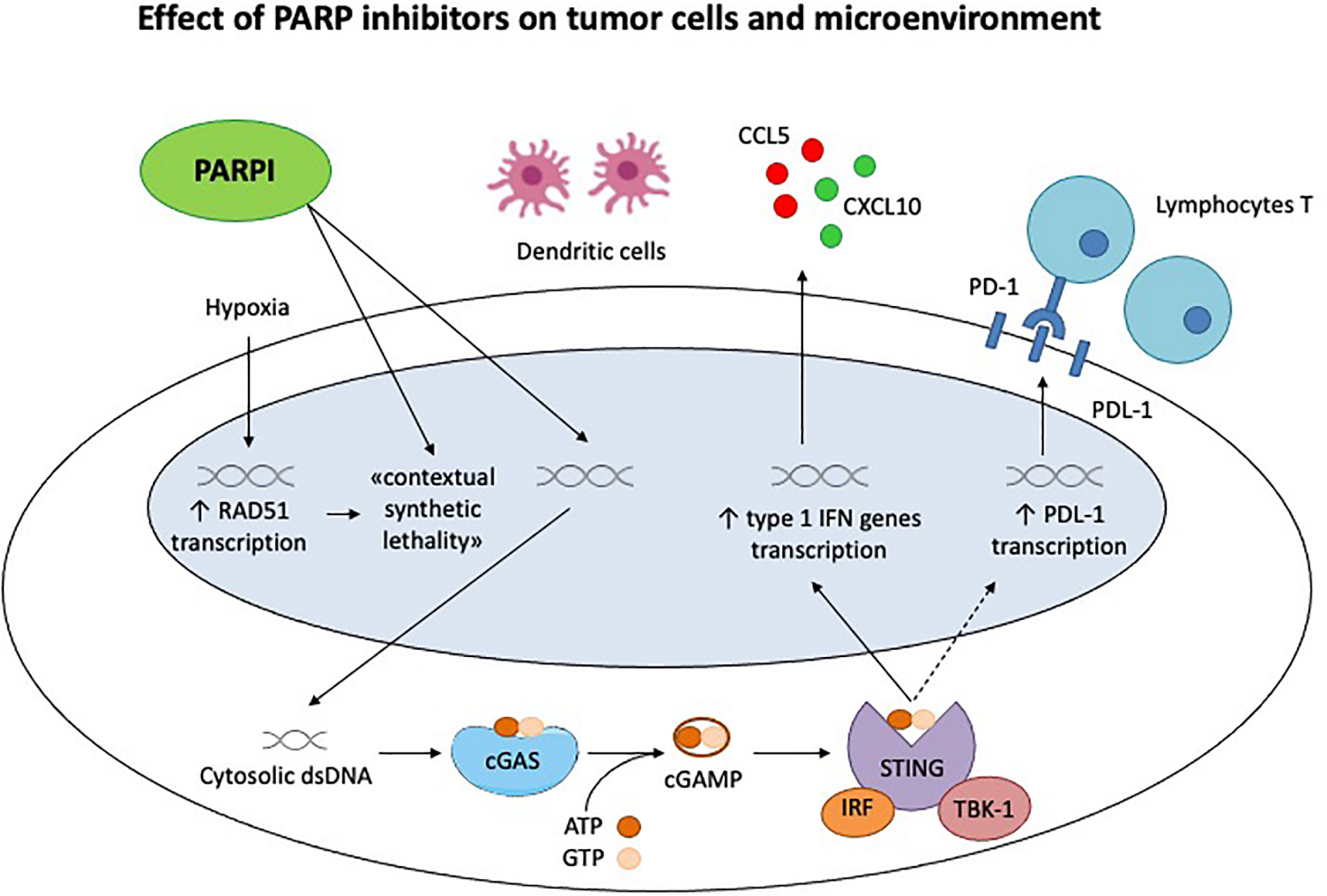
Effect of PARP inhibitors on tumor cells and microenvironment. Chronic hypoxia decreases transcription of homologous recombination proteins, as RAD51, rendering the tumor cells susceptible to PARP inhibitors. This is the concept of «contextual synthetic lethality». Cytosolic double-strand DNA (dsDNA) fragments, generated by the effect of Parpi on tumor cells, activate cGAS-cGAMP-STING pathway, leading to the transcription of type 1 IFN genes and the production of pro inflammatory cytokines. This generates a significant immune response, recruiting activated lymphocytes T and dendritic cells. STING pathway activates also the PDL-1 transcription, inhibiting the immune system through PD-1/PDL-1 interaction.

As discussed in the review, PARPi have numerous ancillary and off target effects; the inhibition of DNA repair and subsequent accumulation of double strand breaks increases the neoantigen load and stimulates the immune system, as well as through the STING pathway (28, 43) (**Figure 1**). In addition to this core action, PARPi have a profound influence on the tumor microenvironment, which in turn is determined by a much more complicated and overlapping series of factors than originally thought, BRCA mutation and HRD being perfect examples (29).

OC has always been considered a poor responder to ICI, but data is now shedding a new light on the matter. Influencing the immune system and the tumor microenvironment *via* the concomitant use of PARPi and other target therapies might be a more successful approach.

So far preclinical studies have produced robust evidence on the association between ICI and PARPi; the combination of anti CTLA4 and PARPi in BRCA1 mutated ovarian cancer models promotes long term responses, due to the local increase of IFNγ in the tumor environment and consequent recruitment and activation of T cells and cytokine production.

The synergistic effects of the combination therapy could be explained by a multiple phases process: firstly, PARP inhibition directly induces tumor cell damage, which increases the neoantigen load and therefore an antitumor T-cell response, a process that is amplified by ICI blockade. Secondly, local activated T cells produce increased levels of IFNγ above a threshold required to enhance the cytotoxic efficacy of PARP inhibition, resulting in additional therapeutic benefit through cell-intrinsic pathways. This indicates that the therapeutic benefit of PARP inhibition can be significantly amplified (17).

This is further proven by the fact that inhibition of T cell recruitment greatly affects response rates to Olaparib in models, suggesting how the benefit is not only dependent on parp depletion but at least partly due to the adaptive immune system (52).

Another very important point is that many preclinical and clinical trials are highlighting how heterogenous OC is, even BRCA1 mutation compared to BRCA2 mutation seem to create different characteristics and therefore different responses to therapies.

Currently, a high tumor mutational burden (TMB) is one of the hallmarks of ICI responders. Growing data points to a correlation between TMB and DDR deficiency, which might explain why BRCA mutated tumors have a higher response rate to ICI and show a stronger immune activation when treated with PARPi, as BRCA is one of the key components of HR; although it needs to be further researched, HRD appears to be a promising predictive biomarker for response to ICI + PARPi (53).

Many clinical trials regarding ICI + PARPi in ovarian cancer are ongoing (**Table 1**), a few with already available and promising data. In a phase II study, in heavily pretreated mostly platinum resistant patients, Durvalumab + Olaparib yielded a 53% disease control rate at 4 months, with good tolerance (54).

**Table 1:** Ongoing trials regarding ICI + PARPi in ovarian cancer.

Clinical trial identifier	Phase	Agents	Design	Status	Outcome
NCT02571725	Phase I	Tremelimumab + Olaparib	BRCAm ROC	Results published	No DLT or grade 3 AE ORR 100%
NCT02484404	Phase II	Durvalumab + Olaparib	Platinum-resistant ROC	Results published	ORR 14% Acceptable toxicity
TOPACIO NCT02657889	Phase II	Pembrolizumab + Niraparib	Platinum-resistant ROC	Results published	ORR 18% PFS 3.4 months Acceptable toxicity
MEDIOLA NCT02734004	Phase II	Durvalumab + Olaparib	BRCAm platinum-sensitive OC	Results published	ORR 63% Acceptable toxicity
NCT04034927	Phase II	Tremetinib + Niraparib	Platinum-sensitive advanced OC	Recruiting	
KEYLYNK-001 NCT03740165	Phase II	Pembrolizumab + Niraparib	BRCAwt advanced OC	Recruiting	
NCT02484404	Phase I-II	Durvalumab + Niraparib	Advanced, recurrent, or metastatic ovarian, triple negative breast, lung, prostate, colorectal carcinoma or solid tumors	Recruiting	
DUO-O NCT03737643	Phase III	Durvalumab + Niraparib	Advanced OC	Recruiting	
GUIDE2REPAIR NCT04169841	Phase II	Durvalumab + Tremelimumab + Niraparib	HRR-mutated advanced or metastatic solid tumors	Not yet recruiting	
NCT02953457	Phase I-II	Durvalumab + Tremelimumab + Niraparib	DDR-mutated recurrent or refractory OC	Recruiting	
FIRST NCT03602859	Phase III	Durvalumab + Niraparib	Stage III or IV non-mucinous OC	Recruiting	
MOONSTONE NCT03955471	Phase II	Dostarlimab + Niraparib	Advanced platinum-resistant OC	Recruiting	
NCT03806049	Phase III	Dostarlimab + Niraparib	Advanced or recurrent platinum-sensitive OC	Not yet recruiting	
NCT03695380	Phase I	Atezolizumab + Niraparib	Advanced OC	Recruiting	
ANITA NCT03598270	Phase III	Atezolizumab + Niraparib	Recurrent OC	Recruiting	
JAVELIN NCT03642132	Phase III	Avelumab + Niraparib	Advanced OC	Not recruiting	
NCT02873962	Phase II	Nivolumab + Rucaparib	Relapsed OC	Recruiting	
ATHENA NCT03522246	Phase III	Nivolumab + Rucaparib	Advanced OC	Recruiting	
ARIES NCT03824704	Phase II	Nivolumab + Rucaparib	Platinum treated advanced OC	Recruiting	
NCT03101280	Phase I	Atezolizumab + Rucaparib	Advanced or metastatic platinum-sensitive ovarian or endometrial cancer or triple negative breast cancer	Not recruiting	
NITCHE NCT04679064	Phase II	Dostarlimab + Niraparib	Recurrent resistant OC not fit for platinum	Recruiting	

The MEDIOLA basket trial (55) assessed Durvalumab + Olaparib in BRCA mutated OC, breast cancer, and gastric cancer; ovarian cancer patients were platinum sensitive and achieved an 81% disease control rate (DCR) at 12 weeks; furthermore, patients with fewer previous therapy lines had better outcomes. A triplet cohort, adding Bevacizumab to Olaparib and Durvalumab in non-germinal BRCA mutated platinum sensitive OC, was later added. The first results have recently been presented and are very promising, with 77.4 *vs* 28.1% DCR at 24 weeks when compared to the duplet cohort, and similar side effects.

In the TOPACIO trial, platinum resistant OC and triple negative breast cancer patients were treated with Niraparib + Pembrolizumab. ORR was 25%, increasing to 45% in the BRCAmut population.

So far, the data is complex and at times contradictory, which clearly shows the need for further research and for further differentiation of the tumor genetic and environmental signature. This will eventually allow the identification of more accurate predictive biomarkers; this is currently an unmet need, and it highlights how important translation endpoints are in clinical trials in order to bring forward a critical change in patient outcomes; finding reliable predictive markers in such a heterogeneous cancer type is key to correctly selecting patients who will benefit from ICI + PARPi therapy, and to identify strategies to counteract primary and secondary resistance.

## Author Contributions

MT: conceptualization, drafting of the manuscript, and final approval. GS: conceptualization, review and editing, and final approval. VT: review and editing and final approval. GG: review and editing and final approval. GV: conceptualization, supervision, and final approval. All authors contributed to the article and approved the submitted version.

## Funding

This article was partially funded by Italian Ministry of Health, Ricerca Corrente 2021, FPRC 5xmille 2015 MIUR, progetto FUTURO to GV.

## Conflict of Interest

The authors declare that the research was conducted in the absence of any commercial or financial relationships that could be construed as a potential conflict of interest.
